# NEISSERIA MENINGITIDIS PERITONITIS SEROTYPE C: CASE REPORT

**DOI:** 10.1590/0102-6720201600010019

**Published:** 2016

**Authors:** João Kleber de Almeida GENTILE, Maurice Youssef FRANCISS, Hamilton Ribeiro BRASIL

**Affiliations:** 1 Department of General Surgery of the Irmandade da Santa Casa de Misericórdia de São Paulo, São Paulo, SP; 2 Guarulhos General Hospital, Guarulhos, SP, Brazil

## INTRODUCTION

The meningococcal disease manifestation as acute abdomen with meningococcal peritonitis is rare. Is reported primary peritonitis and bacteremia by*Neisseria meningitidis* serotype C occurring in conjunction with the obstructive acute abdomen.

## CASE REPORT

Man with 27 year old was admitted with diffuse abdominal pain accompanied by stop in eliminating flatus and feces for three days and fever 38,3º C for 24 h. As history, had passed prior laparotomy seven years ago for acute appendicitis. He denied other symptoms, recent travel or infectious diseases. There was no recent use of medications or hospitalization. Denied alcohol or illicit drugs.

On examination, he was confused, agitated, dehydrated with clinical signs of sepsis. Was febrile (38,3º C), with tachycardia (112 beats per minute), tachypnea (20 breaths per minute) and hypotension (90x50 mmHg). The abdomen had prior infraumbilical laparotomy scar, very distended, painful diffusely, hypertimpanic and positive to sudden decompression. There was no evidence or clinical signs of liver disease or ascites. Rectal touch was normal without bleeding or mucus in the stool.

Initial investigation showed leukocytosis (18,600 leukocytes with 11% rod cells), metabolic acidosis signals, high C-reactive protein (38.6 mg/l) and abdominal radiography with air-fluid levels without pneumoperitoneum. Abdominal CT scan showed only distension and small amount of free fluid in the abdominal cavity; urinalysis and electrolytes unchanged. Differential diagnoses were acute inflammatory abdomen with diffuse peritonitis and acute obstructive abdomen.

Patient received treatment with appropriate volume expansion 20 ml/kg and antibiotic therapy with ciprofloxacin 400 mg 12/12 h and metronidazole 500 mg 8/8 h. It was referred to explorative laparotomy as urgency after 24 h after admission.

The intraoperative findings were only distension of the small bowel with the presence of thick flanges and thick purulent fluid in the abdominal cavity and pelvis. In the inventory of the cavity was not observed organized abscess and visceral perforation with no identifiable cause for the origin of pus. It was held lysis of adhesions and collection of purulent fluid to culture. The result of the culture was positive for*Neisseria meningitidis* group C, confirmed by polymerase chain reaction. The antibiogram was sensitive to ceftriaxone, meropenem and rifampicin.

Evolved on the 2^nd^ day after surgery with worsening of confusion and positive meningeal signs besides diffuse petechiae and thrombocytopenia (88,000 platelets/mm^3^). Spinal liquor resulted also be positive for *Neisseria meningitidis* group C (diplococci gram negative) with 33,000 cells/mm^3^ (up to 5 cells/mm^3^) 79% of neutrophils, 6 red blood cells (to 0/mm^3^), total protein 172 mg/dl (up to 40 mg/dl) glucose and 1 mg/dl (40-80 mg/dl). It was referred to ICU with diagnosis of meningitis with meningococcemia; began treatment with ceftriaxone 1 g 12/12 h, resulting in improvement of neurological and abdominal symptoms after 72 h.

## DISCUSSION

*Neisseria meningitidis*, Gram-negative diplococcus, was described in 1887 as major cause of meningitis and meningococcal bacteremia in all ages. The dissemination occurs through the nasopharynx with hematogenous spread to the meninges or other organs. It is not part of the normal gastrointestinal flora and isolated only in rectal secretions in combination with sexual transmission. Meningococcal spontaneous peritonitis have been reported in patients with preexisting ascites, but still little understood in patients without liver disease.

The first case was described in 1917 by Moeltoen^4^ and the second with characteristics with appendiceal abscesses, was reported in 1938 by Turchetti^5^. In all cases, the peritonitis is associated with meningococcal disease in other distant sites.

Kelly in 2004 reported a case of peritonitis by *N. meningitidis*diagnosed after laparotomy^3^ similar to acute peritonitis. The theory that can explain the pathophysiological mechanism for this condition is the spread of bacteria through the blood; however, patients with ascites and liver bacterial translocation can justify the isolation of bacteria in peritoneum^1^^,^ 2 ^,^ 3 ^,^ 6.
